# Microsatellite loci in the tiger shark and cross-species amplification using pyrosequencing technology

**DOI:** 10.7717/peerj.2205

**Published:** 2016-08-30

**Authors:** Natália J. Mendes, Vanessa P. Cruz, Fernando Y. Ashikaga, Sâmia M. Camargo, Claudio Oliveira, Andrew N. Piercy, George H. Burgess, Rui Coelho, Miguel N. Santos, Fernando F. Mendonça, Fausto Foresti

**Affiliations:** 1 Laboratório de Biologia e Genética de Peixes, Universidade Estadual Paulista, Botucatu, São Paulo, Brazil; 2 Laboratório de Genética Pesqueira e Conservação, Institute of Marine Sciences, Universidade Federal de São Paulo, Santos, São Paulo, Brazil; 3 Lake Nona Campus, Valencia College, Orlando, Florida, United States; 4 Florida Museum of Natural History, Florida Program for Shark Research, University of Florida, Gainesville, Florida, United States; 5 IPMA, Portuguese Institute for the Ocean and Atmosphere, Algarve, Portugal

**Keywords:** High-throughput sequencing, *Galeocerdo cuvier*, Shark, Microsatellites, Population structure

## Abstract

The tiger shark (*Galeocerdo cuvier*) has a global distribution in tropical and warm temperate seas, and it is caught in numerous fisheries worldwide, mainly as bycatch. It is currently assessed as near threatened by the International Union for Conservation of Nature (IUCN) Red List. In this study, we identified nine microsatellite loci through next generation sequencing (454 pyrosequencing) using 29 samples from the western Atlantic. The genetic diversity of these loci were assessed and revealed a total of 48 alleles ranging from 3 to 7 alleles per locus (average of 5.3 alleles). Cross-species amplification was successful at most loci for other species such as *Carcharhinus longimanus, C. acronotus* and *Alopias superciliosus*. Given the potential applicability of genetic markers for biological conservation, these data may contribute to the population assessment of this and other species of sharks worldwide.

## Introduction

The tiger shark *Galeocerdo cuvier* (Péron & Lesueur, 1822), is a shark from the order Carcharhiniformes and family Carcharhinidae. It has a worldwide distribution in tropical and temperate seas, and is considered a top predator generally requiring large foraging areas (Heupel et al., 2014). Recent data shows that this species can move long distances and occupies different regions, including coastal areas being therefore more susceptible than pelagic sharks to anthropogenic threats (Heupel et al., 2014).

Caught in many world fisheries as bycatch, the tiger shark is currently classified as “Near Threatened” by the International Union for Conservation of Nature (IUCN). However, some basic information, such as the characterization of population genetic structure, variability, identification of geographical restrictions to gene flow with possible local populations remains broadly unknown. In light of this, information on their conservation status is difficult to assess. For this reason, molecular markers have been increasingly used in species conservation and management programs, including microsatellite molecular markers (Simple Sequence Repeats–SSR). A range of SSR markers have been developed using the pyrosequencing technique, generating information with millions of base pairs in a single run and in a short period of time.

Specifically for the tiger shark, nine SSR markers were previously developed on specimens from the Hawaiian archipelago (Bernard, Feldheim & Shivji, 2015), but cross-application was not tested for other shark species yet. Thus, the objectives of this study were to identify other microsatellites for the tiger shark in specimens from the Atlantic, and design additional molecular markers that can now be used in this and other shark species for population genetics studies.

## Material and Methods

### Sampling

In fulfillment of data archiving guidelines (Baker, 2013), primary data have been deposited with Dryad. Samples of tiger shark were collected in landings of the fishing fleet from São Paulo coast (n = 12) and in scientific cruises in the Fernando de Noronha archipelago (n = 6) by researchers from the Biosciences Institute of Botucatu, São Paulo State University, and Marine Sciences Institute of the São Paulo Federal University, in Brazil. Additionally, 11 samples were collected from the east coast of Florida by the Florida Program for Shark Research, University of Florida, USA. For evaluating cross-amplification we used six samples of *Carcharhinus acronotus* collected from São Paulo coast, five samples of *C. longimanus* and five samples of *Alopias superciliosus*, collected in the northeast Atlantic by onboard observers from the Portuguese Institute for the Ocean and Atmosphere (IPMA), Portugal. All sampled tissues were stored in 95% ethanol to ensure the integrity and quality of tissue for molecular analysis.

### 454 GS-FLX pyrosequencing and microsatellite discovery

The total genomic DNA was extracted from each sample following the protocol described by Ivanova, Dewaard & Hebert (2006). A sample (voucher: TIG03SP) of 100 μg of tiger-shark DNA from São Paulo coast (−25.1164, −47.6082) was sequenced on a Roche 454 GS FLX sequencer with Titanium platform “Genome sequencer 20 System” (Instituto Agrobiotecnológico de Rosário–INDEAR, Argentina), following procedures described in Margulies et al. (2005).

To isolate microsatellites and design primers for population genetics all sequences of the SSR were compiled using Primer3 (Rozen & Skaletsky, 1999) and BatchPrimer3 (You et al., 2008). Primers were designed based on the following criteria: primer size of 20 bp (min = 18, max = 22 bp), ideal annealing temperature of 60 °C (min = 55 °C, max = 63 °C), GC optimum of 60% (min = 40%, max = 80%) and the size of the amplified product ranging from 50–500 bp. The sequences were then grouped and aligned in the Clustal W software (Thompson, Higgins & Gibson, 1994), identifying duplicated sequences for the same locus.

### Novel microsatellite markers

The PCR amplifications to test the synthesized primers were performed in a Thermal Cycler Veriti™ (Applied Biosystems, Foster City, CA, USA) under the following conditions: initial denaturing for 10 min at 95 °C; 30 cycles of 94 °C for 45 s, the primer annealing temperature (TA) was tested from of 51 to 57 °C for 50 s; 72 °C for 50 s, and a final extension at 72 °C for 20 min. The total reaction volume was 10 μL and composed of 1.0× PCR Buffer, 0.25 mM MgCl_2_, 0.05 mM of each dNTP, 0.5 units of Platinum Taq DNA polymerase (InvitrogenCarlsbad, CA, USA), 0.10 μM reverse primer, 0.10 μM forward primer, and 30 ng of template DNA.

To verify the effectiveness of the reaction and the amplification of the fragments, 1.5 μL of the PCR product were subjected to electrophoresis on a 1% agarose gel. The amplified products were compared with a 1 Kb plus ladder (Invitrogen), subsequently visualized on a transilluminator and photographed with a digital camera using the *Kodak Digital Science software*.

Genotyping was done with the M13-tail PCR method of Schuelke (2000). The best loci, that showed high polymorphism and quality of bands, were selected and further analyzed on an ABI 3130xl sequencer (Applied Biosystems, Life Technologies). The allele sizes were determined using ROX 500 (Applied Biosystems) as an internal standard with the software package *GeneMapper 3.7* (Applied Biosystems).

We used the software GenAlex analysis 6.1 (Peakall & Smouse, 2006) to convert our data to run in other analysis programs. Arlequin 3.5 (Excoffier & Lischer, 2010) was used to calculate heterozygosity, number of alleles, Hardy-Weinberg equilibrium (HWE) and linkage disequilibrium. The program *Cervus v.3.0.7* (Marshall et al., 1998) was used to test for the presence of null alleles and estimate, the inbreeding coefficient (Fis) and polymorphism information content (PIC).

## Results and Discussion

From the genomic material generated by the pyrosequencing technology, a total of 71,059 reads with an average size of 367 bp was obtained, consisting of 26,075,405 nucleotides, which accounts for approximately 0.75% of the *G. cuvier* genome, assuming a genome size of 3.44 Gb (estimated from the size of *Rhincodon typus*, Read et al., 2015). For the identification of microsatellite sequences, the online software *Batch Primer3* was used, and 615 microsatellite loci were identified. A second filtration was subsequently performed with the software *Primer 3.0* which identified 159 microsatellite loci. From these, we selected 30 loci which contained the best scores of each primer pair with a size of 15–20 bp, a GC of 40–50% and little variation in the annealing temperature in the PCR reaction. Of these, 20 pairs of primers were synthesized and tested, nine being polymorphic with one trinucleotide and eight dinucleotide primers (Table 1). The sequences for polymorphic microsatellite markers in this study have been deposited in GenBank (Accession numbers: KT598263–KT598271).

**Table 1:** General information about the microsatellite analysis.

Analyzes	N of sequences
Number of reads	71.059
Selection of microsatellites (using BatchPrimer3)	615
Secondary selection of microsatellite (using Primer 3.0)	159
Amplification and control of PCR product on agarose gel	30
Microsatellite loci to synthesize with fluorescent dye	20
Polymorphism test with capillary sequencer	10
Microsatellite loci in linkage equilibrium	9

The application of the developed markers resulted in 48 alleles, with a minimum of 3 (TIG_25) to 7 (TIG_1, TIG_7, TIG_12) and average of 5.3 alleles per loci. Transferability tests of the markers in other species showed positive amplification in *C. longimanus, Alopias superciliosus* and *C. acronotus*. For the *C. acronotus*, two loci were polymorphic (TIG_17, TIG_5), and for *C. longimanus* and *Alopias superciliosus* only one polymorphic locus were observed in five samples of each species, TIG_15 and TIG_7, respectively (Table 2).

**Table 2:** Data for microsatellite loci of the cross-amplification in *Carcharhinus longimanus, Carcharhinus acronotus* and *Alopias superciliosus*.

*C. acronotus*			*C. longimanus*		*A. superciliosus*	
Loci	Na	Range (bp)	Na	Range (bp)	Na	Range (bp)
TIG_1	2	116–118	2	118–134	2	104–118
TIG_5	3	260–264	2	331–335	2	265–273
TIG_7	2	170–180	2	162–170	3	152–170
TIG_10	2	251–253	1	304	1	307
TIG_12	2	296–364	2	246–296	2	372–418
TIG_15	1	336	3	290–310	2	288–312
TIG_17	3	242–270	2	210–224	1	268
TIG_19	0	0	1	316	2	386–394
TIG_25	1	396	1	358	2	388–398

**Note:**

Na, number of alleles.

**Table 3:** Data for microsatellite loci in the tiger shark, *Galeocerdo cuvier*.

Loci	Primer sequence (5′→3′)	MOTIF	T °C	N	Na	Range (bp)	Ho	He	HWE	Fis	PIC	F (Null)
TIG_1	F_CTCTTGACGGTGCTCGATC	(AC)10	53	29	7	116–154	0.758	0.642	0.711	−0.184	0.710	−0.194
	R_AATGGCAACTTTTCCTGTCC											
TIG_5	F_GCCAGCATCCATTCATACAG	(CT)8	51	26	4	203–257	0.384	0.337	1.000	−0.141	0.589	−0.239
	R_AGAGGGAAGTGGTGTGTGGT											
TIG_7	F_CACCAACCTCCCCATCAC	(AC)15	57	27	7	169–183	0.925	0.726	0.318	−0.280	0.811	−0.101
	R_CAGACATTCCTCCTCCATCC											
TIG_10	F_CTCAGCAGGTCTGGACAACA	(GT)10	59	29	5	256–276	1.000	0.655	0.000	−0.539	0.608	−0.245
	R_GGTGGTAGGAACATGGAACG											
TIG_12	F_TGCCATGAGTGCTGTTTTTC	(CA)11	53	28	7	364–376	0.535	0.520	0.543	−0.030	0.682	−0.213
	R_TGCCGCATTGTTACTGCTAC											
TIG_15	F_AACTGCCAAAAGGGACAAGA	(TG)15	55	25	6	231–241	0.520	0.463	0.675	−0.124	0.650	−0.233
	R_GTAAGCCCAACAGACCATCC											
TIG_17	F_TGAAGCTAACGAGGGGTCTG	(GT)11	57	25	4	268–286	0.160	0.554	0.000	0.715	0.734	−0.138
	R_AGCGCAGAAGATCAAGAGGA											
TIG_19	F_TGCTTGTGTCTGAGGTGAGTG	(TG)10	53	27	5	337–353	0.555	0.443	0.677	−0.260	0.627	−0.214
	R_TTGGAGGTTCAATCCGAGAC											
TIG_25	F_CCGTGCCTATGTGGATTTCT	(CCT)5	55	27	3	331–349	0.222	0.206	1.000	−0.075	0.511	−0.285
	R_CTTGAAGAGAGTGGGCGAAG											

**Notes:**

T °C, primer annealing temperature; N, number of individuals analyzed; NA, number of alleles; He, expected heterozygosity; Ho, observed heterozygosity; HWE, probability of departure from Hardy-Weinberg equilibrium; Fis, inbreeding coefficient; PIC, polymorphism information content; F (Null), null alleles.

In tiger shark the observed heterozygosity (Ho) and expected heterozygosity (He) ranged from 0.16 (TIG_17) to 1.0 (TIG_10) and 0.20 (TIG_25) to 0.72 (TIG_7), respectively (Table 3). The Ho was higher than He, suggesting an excess of heterozygotes relative to the model of HWE. Significant differences from HWE after Bonferroni correction (p < 0.01) were detected in only two loci (TIG_10 and TIG_17). The deviation in the HWE for locus TIG_17 (0.715) can be explained by a significant value in intrapopulation Fis (Kordicheva et al., 2010). This locus was the only one with a positive value for the Fis, and may be evidence of a heterozygous deficiency (Holsinger & Weir, 2009), resulting in a decrease in genetic variability.

Imbalance values in Hardy-Weinberg equations when considering microsatellite locus may be due to the presence of null alleles (Kordicheva et al., 2010). However, the presence of null allele was not detected in the present study, indicating that the markers developed are of high quality. Further, the PIC was highly informative for all the loci (PIC > 0.5), also indicating a high quality marker (Botstein et al., 1980).

In the present study, the average expected heterozygosity was approximately 0.50 and the average observed heterozygosity was 0.55. The levels of genetic variability seen in this study may be due to population differences resulting from remote sample locations. This is to be expected given that the samples are coming from different oceans and the finding of significant differences in the levels of heterozygosity among different groups would not be unforeseen.

## Additional Information and Declarations

### DNA Deposition

The following information was supplied regarding the deposition of DNA sequences: GenBank BankIt1851998 Seq1 KT598263 BankIt1851998 Seq2 KT598264 BankIt1851998 Seq3 KT598265 BankIt1851998 Seq4 KT598266 BankIt1851998 Seq5 KT598267 BankIt1851998 Seq6 KT598268 BankIt1851998 Seq7 KT598269 BankIt1851998 Seq8 KT598270 BankIt1851998 Seq9 KT598271.
